# Performance characteristics of chitosan ε-polylysine composite films and their application in tuna shelf-life extension

**DOI:** 10.3389/fnut.2026.1766857

**Published:** 2026-02-16

**Authors:** Yiran Lu, Ye Tao, Supaluck Kraithong, Jiayue Fang, Hao Lan, Jingrong Gao, Yadong Zhao, Shanggui Deng

**Affiliations:** 1 School of Food and Pharmacy, Zhejiang Ocean University, Zhoushan, Zhejiang, China; 2 Guangxi Key Laboratory of Marine Drugs, Institute of Marine Drugs, Guangxi University of Chinese Medicine, Nanning, Guangxi, China; 3 College of Food Science and Technology, Nanjing Agricultural University, Nanjing, Jiangsu, China

**Keywords:** antioxidant, fish freshness, myoglobin degradation, spoilage indicators, ε-polylysine and chitosan

## Abstract

**Introduction:**

Yellowfin tuna (*Thunnus albacares*) is highly susceptible to microbial spoilage, lipid oxidation, and myoglobin (Mb) oxidation during refrigerated storage, which severely limits its shelf life. The development of natural, multifunctional preservation systems is therefore essential for maintaining tuna quality.

**Methods:**

Chitosan-ε-polylysine (CS-ε-PL) composite films with different ε-PL concentrations were applied to refrigerated yellowfin tuna. Preservation efficacy was systematically evaluated by monitoring Mb oxidation, microbial growth, lipid oxidation, nucleotide degradation, color stability, and biochemical spoilage indicators during storage at 4 °C.

**Results:**

The CS-ε-PL composite films significantly delayed quality deterioration compared with untreated controls. ε-PL markedly inhibited Mb oxidation, with the ε-PL-0.15 group retaining 49.1 g/kg metmyoglobin (Met) by day 8, nearly double that of the control group (26.5 g/kg). Lipid oxidation (TBA: 0.55 vs. 0.73 mg/100 g) and nucleotide degradation (*K* value: 22.3 vs. 36.7) were substantially suppressed, while color stability was better maintained (a^*^ value: 5.79 vs. 2.97). The accumulation of total volatile basic nitrogen (TVB-N) was reduced by 43% (25.6 vs. 44.7 mg/100 g). Moreover, microbial growth was effectively controlled, with total viable counts limited to 6.3 log CFU/g compared with 7.8 log CFU/g in the control group.

**Discussion:**

The enhanced preservation performance of CS-ε-PL films is attributed to the synergistic effects between the physical barrier properties of chitosan and the broad-spectrum antibacterial and antioxidant activities of ε-polylysine. This multifunctional composite system effectively retards oxidative reactions and microbial proliferation, thereby significantly extending the refrigerated shelf life of yellowfin tuna.

## 1 Introduction

Yellowfin tuna (*Thunnus albacares*) is a high-value seafood commodity renowned for its unique organoleptic properties and rich nutritional components (1). It is widely distributed in China, the South China Sea, the East China Sea, and along the coast of Taiwan (2), and its post-harvest preservation has long been a critical concern for the seafood industry. The species' intrinsic biological characteristics—particularly its high myoglobin (Mb) content and abundant iron-binding proteins—make it highly susceptible to oxidative discoloration, textural degradation, and flavor loss, leading to rapid freshness decline and significant economic losses (3).

The deterioration of yellowfin tuna is driven by multiple, interconnected mechanisms. Mb, as the predominant pigment in tuna muscle, is responsible for the characteristic red color and oxygen storage function (4). Post-harvest oxidation of Mb impairs its oxygen-binding capacity, causing a color shift from bright red to brown (5). Concurrently, protein degradation and microbial metabolic activity lead to the accumulation of total volatile basic nitrogen (TVB-N), a key spoilage indicator (6). Microbial proliferation, particularly by species such as *Pseudomonas fluorescens* and *Bacillus subtilis*, further accelerates off-flavor formation and textural deterioration (7, 8). Lipid oxidation and nucleotide degradation also synergistically exacerbate quality loss, highlighting the multifaceted nature of tuna spoilage (9). Traditional preservation methods such as refrigeration and freezing can slow but not fully prevent these deterioration processes (10).

Natural biopolymers have emerged as promising alternatives for seafood preservation due to their biodegradability, non-toxicity, and functional versatility (11). Among these, chitosan and ε-polylysine (ε-PL) have been extensively studied for their antimicrobial, antioxidant, and film-forming properties (12–14). Chitosan, a polysaccharide derived from crustacean exoskeletons, inhibits microbial growth through membrane disruption and forms protective films that reduce oxygen exposure, limiting oxidation (15). However, its preservation efficacy diminishes over time, particularly under the oxidative stress associated with high-Mb fish (11). ε-PL complements chitosan by providing broad-spectrum antimicrobial activity against common spoilage bacteria and exhibiting antioxidant effects that reduce lipid oxidation, thereby helping maintain fish color and flavor (8, 16). Composite biopolymer systems have shown synergistic enhancement of preservation, for example, chitosan-based films modified with functional additives can inhibit lipid oxidation, protein degradation, and biogenic amine accumulation (17).

Despite these advances, the application of chitosan-ε/-PL composites to address the unique spoilage challenges of high-Mb fish like yellowfin tuna remains underexplored. Specifically, systematic evaluation of their synergistic effects on Mb oxidation, iron-mediated lipid peroxidation, protein degradation, and microbial growth has not been conducted. Therefore, the present study aims to develop and characterize chitosan-ε-PL composite films and assess their efficacy in preserving refrigerated yellowfin tuna. By monitoring key indicators such as TBA, TVB-N, and microbial load, this work seeks to elucidate the structure–function relationships underlying preservation effects. The innovation lies in integrating chitosan's barrier properties with ε-PL's antimicrobial and antioxidant activities to provide a natural, effective, and targeted strategy for extending the shelf life of high-Mb seafood.

## 2 Materials and methods

### 2.1 Raw materials

Fresh yellowfin tuna (*Thunnus albacares*) was sourced from a local fish market, selected for freshness and uniform size to ensure experimental consistency. Processed into 2 cm × 2 cm muscle slices within 6 h of arrival, samples were stored on ice at 4 °C until use. Chitosan: food-grade (deacetylation degree 85%, purity ≥95%), Shanghai Macklin Biochemical Technology Co., Ltd., China. ε-Polylysine (ε-PL): pharmaceutical-grade (purity ≥98%, derived from Streptomyces), Sigma-Aldrich (USA) or Shanghai Yuanye Biotechnology Co., Ltd., China. Dissolved in 1% glacial acetic acid (analytical grade, ≥99.5%, Xilong Technology Co., Ltd., China) to prepare 0.1% and 0.15% (w/v) film-forming solutions. Thiobarbituric acid (TBA) kit: Nanjing Jiancheng Bioengineering Institute, China (meets food chemistry detection standards). Potassium dihydrogen phosphate (analytical grade, ≥99.0%, Sinopharm Chemical Reagent Co., Ltd., China) was used to prepare 0.02 mol/L pH 4.0 buffer. Methanol (chromatographic grade, ≥99.9%, Sinopharm Chemical Reagent Co., Ltd., China) was mixed with the buffer (95:5, v/v) as HPLC mobile phase. Physiological saline (medical grade, 0.9% NaCl, Baxter Healthcare Co., Ltd., China) was used for microbial count sample dilution. Boric acid solution (analytical grade, ≥99.0%, Tianjin Kemiou Chemical Reagent Co., Ltd., China), standard hydrochloric acid (1 mol/L, calibrated, Shanghai Titan Technology Co., Ltd., China), and magnesium oxide (analytical grade, ≥98.0%, Tianjin Benchmark Chemical Reagent Co., Ltd., China) were used for TVB-N determination. Other reagents were commercial products meeting food science experimental grade requirements.

### 2.2 Experimental equipment

Tensile testing machine: Instron 5967 (Instron, USA) for film TS and EAB; Colorimeter: Konica Minolta CR-400 (Japan) for L^*^, a^*^, b^*^ measurement of films and tuna; FT-IR: Nicolet iS50 (Thermo Fisher, USA) with scanning range 4000–600 cm^−1^ for functional group analysis; SEM: Hitachi SU8010 (Japan) at accelerating voltage 10 kV for film cross-sectional morphology; XRD: Bruker D8 Advance (Germany) with 2θ 5°-50° and scanning rate 4°/min for film crystallinity; TGA: NETZSCH TG 209 F1 Libra (Germany) with 30 °C−600 °C heating range and 10 °C/min rate (N_2_ atmosphere) for thermal stability; HPLC: Shimadzu LC-20AT (Japan) equipped with VP-ODS C18 column (4.6 mm × 250 mm, 5 μm) for *K* value; Spectrophotometer: Shimadzu UV-1800 (Japan) for myoglobin content, TBA value and absorbance assays; Centrifuge: Eppendorf 5810R (Germany) at 4 °C and 10,000 r/min for sample centrifugation; Homogenizer: IKA T18 basic (Germany) for tuna muscle homogenization; Incubator: Thermo Scientific HERACell 150i (USA) for microbial culture (30 °C/48 h, 7 °C/10 days); Kjeldahl apparatus: KDY-9820 (Beijing Ruibang Xingye, China) for TVB-N determination.

### 2.3 Preparation of composite film

Control Group (Untreated): fresh tuna samples were stored under refrigeration conditions, and wrapped with commonly available cling film on the market, recorded as CK group.

Chitosan: dissolve 1.5 g of chitosan in 100 ml of 1% acetic acid solution to prepare a film-forming solution. Then pour the solution into a culture dish and place it in a well ventilated area to air dry for 24 h. After the drying process is completed, an edible film is formed. Subsequently, the fish slices were wrapped with the film, and this treatment group was called the chitosan group.

ε-PL-0.1 and ε-PL-0.15: dissolve 0.1 g of ε-polylysine and 1.5 g of chitosan in 100 milliliters of 1% acetic acid solution to prepare the ε-PL-0.1 group; Alternatively, 0.15 g of ε-polylysine and 1.5 g of chitosan can be dissolved in 100 milliliters of 1% acetic acid solution to prepare the ε-PL-0.15 group. Pour each solution into a culture dish and air dry in a well ventilated area for 24 h. After pre wetting the membrane, it was used to wrap fish slices and named as the ε-PL-0.1 group and the ε-PL-0.15 group chitosan group, respectively.

After packaging, the samples were placed in sealed bags, and each group was further subdivided according to storage time. The total bacterial count experiments were conducted at intervals of 2, 4, 6, 8, 10, and 12 days, while other experiments were conducted at intervals of 1, 2, 3, 4, 5, 6, 7, and 8 days. The tuna sample is placed in a polyethylene bag and refrigerated at 4 °C ± 1 °C throughout the entire storage period.

### 2.4 Physical properties of single-layer composite film

#### 2.4.1 Determination of physical, mechanical, and color difference

The film thickness was measured using a micrometer at multiple random positions, and the average value was recorded. Water vapor permeability (WVP) was determined using the desiccant method. Films were sealed onto weighing dishes containing anhydrous calcium sulfate and placed in a controlled humidity chamber; the weight gain was monitored over time to calculate WVP.

Mechanical properties were evaluated by measuring tensile strength (TS) and elongation at break (EAB). Film samples were cut into strips (10 mm × 50 mm), clamped in a tensile testing apparatus with an initial grip distance of 30 mm, and stretched at a speed of 0.5 mm/s.

Color parameters (L^*^, a^*^, b^*^) were measured using a colorimeter with a standard white board as reference. The total color difference (ΔE) was calculated to assess visual appearance.

#### 2.4.2 Fourier transform infrared spectroscopy (FT-IR)

The FT-IR spectra of the poly-L-lysine (PL) films were recorded in the range of 4,000–600 cm^−1^. Film samples, cut into 2 cm × 2 cm squares, were placed directly onto the sample holder. The spectra were obtained by averaging 32 scans at a resolution of 4 cm^−1^. The characteristic peaks of the PL films were analyzed to investigate the functional groups present and the interactions between PL and other components in the composite films.

#### 2.4.3 Scanning electron microscopy (SEM)

The surface morphology of the poly-L-lysine films was examined using a scanning electron microscope after coating the samples with a thin layer of gold. The film samples, cut into 1 cm × 1 cm^2^, were adhered to double-sided conductive tape. SEM observations were performed at an accelerating voltage of 10 kV to analyze the surface structure, smoothness, and homogeneity of the films.

#### 2.4.4 X-ray diffraction (XRD)

The crystallization characteristics of the poly-L-lysine films were determined by X-ray diffraction using a diffractometer equipped with a Cu Kα radiation source. The XRD patterns were recorded over a diffraction angle (2θ) range of 5° to 50° at a scanning rate of 4°/min under the conditions of 40 kV and 100 mA. The XRD analysis was used to assess the crystalline structure and phase transitions of the PL films and to compare the effect of different processing conditions on their crystallinity.

#### 2.4.5 Thermogravimetric analysis (TGA)

The thermal stability of the poly-L-lysine films was evaluated using thermogravimetric analysis. Approximately 5 mg of each film sample was placed in an aluminum pan and heated from 30 °C to 600 °C at a heating rate of 10 °C/min under a nitrogen atmosphere to prevent oxidation. The TGA curves were recorded, and the onset degradation temperature, as well as the maximum degradation rate, were determined to assess the thermal stability and degradation behavior of the films.

### 2.5 Quality assessment of yellowfin tuna

#### 2.5.1 Microbial count

Microbial analysis was performed to evaluate the effectiveness of the treatments in inhibiting microbial growth. In terms of Huang's research methodology (18). At each time point, 10 g of tuna muscle was homogenized in 90 mL of sterile saline solution, serially diluted, and plated on nutrient agar. Incubation at 30 °C for 48 h or 7 °C for 10 days (psychrotrophic bacteria), and the total plate count (TPC) was determined by counting the colonies formed. Results were expressed as log CFU/g.

#### 2.5.2 Myoglobin content

Following the method of Viriyarattanasak et al. (5), to determine the myoglobin content in fish meat, accurately weigh 2.00 g of fish meat and place it in a 50 ml centrifuge tube. Add 20 ml of 40 mmol/L phosphate buffer solution (pH 6.8, 4 °C). Homogenize the sample at a speed of 10,000 r/min for 15 s, then centrifuge at 4 °C and 10,000 r/min for 10 min. After taking the supernatant, measure the absorbance at wavelengths of 500, 525, 540, and 555 nm using a spectrophotometer. Using the same buffer as the blank.

R1 = A572/A525

R2 = A565/A525

R3 = A545/A525

MetMb (%) = [1.395–(R_1_×2.706)–(R_2_ × 0.322)–(R_3_×1.588)] × 100 where A_525_, A_545_, A_565_, and A_572_ denote the absorbance values measured at 525, 545, 565, and 572 nm, respectively.

All measurements were performed in triplicate, and the results were expressed as percentage of total myoglobin present in the muscle extracts.

#### 2.5.3 TVB-N measurement

TVB-N levels were determined using the Kjeldahl method. Using an improved method for TVB-N value detection of tuna meat (19). A 5 g portion of the tuna sample was homogenized with 50 ml of distilled water. The sample was heated in a reflux apparatus to distill ammonia into a boric acid solution, and the ammonia was titrated with a standard acid solution. TVB-N concentrations were expressed as mg N/100 g.

#### 2.5.4 TBA value (lipid oxidation)

The TBA value follows Souza's method (20), Lipid oxidation was measured using the thiobarbituric acid reactive substances (TBARS) assay. A 2 g portion of tuna muscle was homogenized in 10 ml of TBA reagent, followed by heating. Absorbance was measured at 532 nm using a spectrophotometer, and TBA values were reported as μmol malondialdehyde (MDA) equivalents per kg of tuna.

#### 2.5.5 Color measurement (a^*^ value)

The color of the tuna samples was measured using a colorimeter (Shenzhen Linshang Technology Co., Ltd., China) to determine the a^*^ value (redness). This parameter provides an indication of the tuna's appearance, with higher a^*^ values corresponding to more intense redness. Measurements were taken at each storage time point.

#### 2.5.6 *K* value (nucleotide degradation)

The *K* value refers to Yokoyama's experimental method (21). Nucleotide degradation was evaluated using the *K* value, which reflects the breakdown of ATP and its derivatives. The method involves measuring the levels of inosine monophosphate (IMP), inosine (IN), and hypoxanthine (Hx) in the tuna muscle samples by high-performance liquid chromatography (HPLC). A C18 chromatographic column was used with a mobile phase of 0.02 mol/L potassium dihydrogen phosphate buffer (pH 4.0): methanol = 95:5 (v/v), and the detection wavelength was 254 nm. The *K* value was calculated by the formula: *K* (%) = [*IMP*] + [*IN*] + [*Hx*][*IN*] + [*Hx*] × 100 after quantifying via standard curves. All measurements were performed in triplicate.

### 2.6 Statistical analysis

All experiments were replicated at least three times, and the data acquired were presented as mean ± standard deviation. Statistical analysis was performed using one-way analysis of variance (ANOVA) and Duncan's multiple range test. IBM SPSS Statistics 20 software was employed for statistical analysis, with the significance level defined at *P* < 0.05.

## 3 Results and discussion

### 3.1 Color difference and physical properties

According to Table 1, the incorporation of ε-polylysine (ε-PL) significantly enhanced the overall performance of the composite films. Compared with the pure chitosan (CS) film (0.134 ± 0.04 mm), the ε-PL composite films (ε-PL-0.1 and ε-PL-0.15) exhibited reduced thicknesses (0.0895 ± 0.07 mm and 0.0943 ± 0.05 mm), consistent with Gan et al. (22), who reported more compact microstructures in ε-PL-incorporated polysaccharide films. Mechanically, the composite films showed markedly increased elongation at break (EAB: 52.4 ± 1.03% to 57.56 ± 0.87%) and a moderate decrease in tensile strength (TS: 13.4 ± 0.21 MPa to 14.7 ± 0.29 MPa) compared to the CS film (EAB: 39.7 ± 0.69%, TS: 18.92 ± 0.23 MPa), aligning with observations by Xu et al. (23) regarding enhanced flexibility. This mechanical trade-off arises from ε-PL-chitosan interactions: electrostatic attraction and hydrogen bonding (validated by FT-IR) disrupt chitosan's crystalline structure (XRD evidence), boosting chain mobility to increase EAB, while diluted hydrogen bonding density weakens intermolecular cohesion, leading to lower TS.

**Table 1:** Basic physical properties of each group of thin films.

**Parameters**	**CS**	**PL-0.1**	**ε-PL-0.15**
Thickness	0.134 ± 0.04^a^	0.0895 ± 0.07^b^	0.0943 ± 0.05^b^
WVP (g·m^−1^s^−1^Pa^−1^)	2.67 × 10^−10^ ± 0.092^a^	2.83 × 10^−10^ ± 0.071^a^	2.94 × 10^−10^ ± 0.054^b^
TS (Mpa)	18.92 ± 0.23^a^	13.4 ± 0.21^c^	14.7 ± 0.29^b^
EAB (%)	39.7 ± 0.69^c^	52.4 ± 1.03^b^	57.56 ± 0.87^a^
L^*^	90.58 ± 0.15^a^	89.07 ± 0.27^b^	87.49 ± 0.37^c^
a^*^	−0.09 ± 0.02^a^	−0.01 ± 0.03^a^	1.41 ± 0.12^b^
b^*^	5.86 ± 0.17^a^	7.58 ± 0.49^b^	14.13 ± 0.61^c^

WVP, water vapor permeability; EAB, elongation at break; TS, tensile strength. Different lowercase letters in the same row meant significant difference (*p* < 0.05) of samples in the independent sample *t*-test.

In terms of water vapor permeability (WVP), the composite films (2.83 × 10^−10^ to 2.94 × 10^−10^ g·m^−1^·s^−1^·Pa^−1^) were slightly higher than the CS film (2.67 × 10^−10^ g·m^−1^·s^−1^·Pa^−1^). As noted by Debeaufort et al. (24), polymer polarity influences WVP, suggesting that the minor increase in permeability may slightly compromise moisture barrier properties but is offset by other functional benefits. For color parameters, the ε-PL-0.15 film exhibited significant changes in a^*^ (1.41 ± 0.12) and b^*^ (14.13 ± 0.61) values compared to the CS film (a^*^: −0.09 ± 0.02, b^*^: 5.86 ± 0.17) (*P* < 0.05), reflecting structural modifications induced by ε-PL as reported by Zhang et al. (25). Overall, ε-PL incorporation improves mechanical flexibility and structural compactness, making these films suitable for tuna preservation despite the slight increase in WVP.

### 3.2 FT-IR

**Figure 1:**
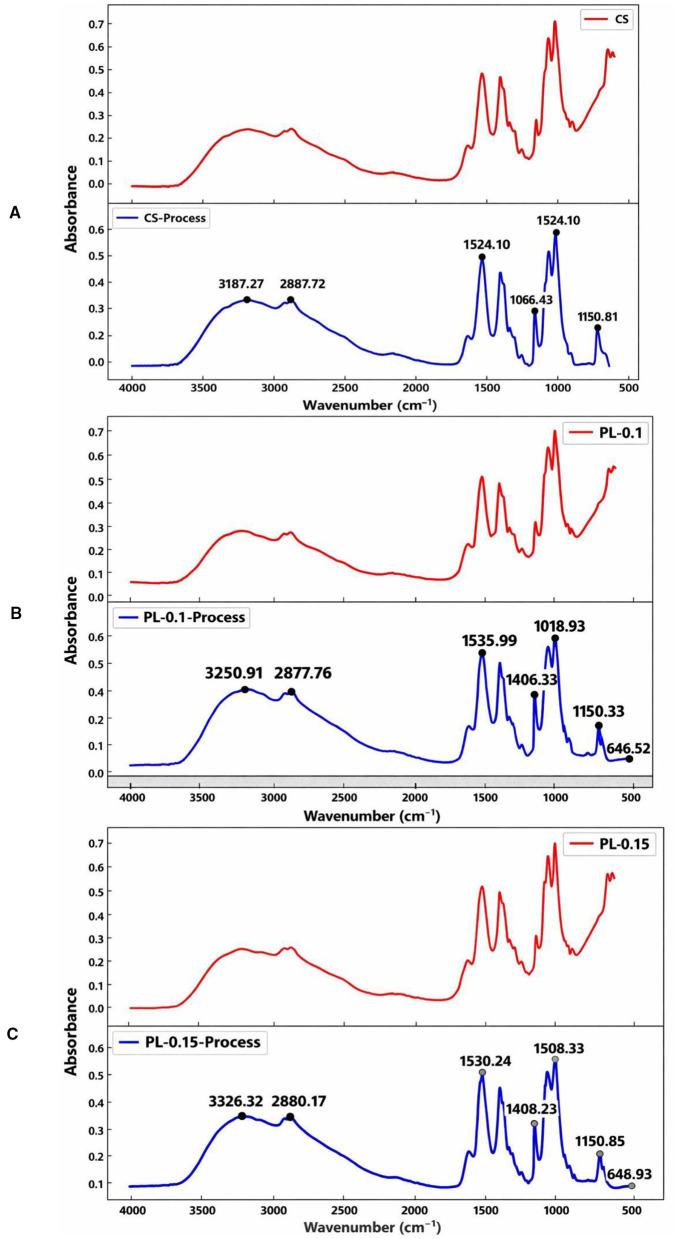
FTIR spectra of different films, CS **(A)**, PL-0.1 **(B)**, ε-PL-0.15 **(C)**.

FT-IR spectra were recorded to analyze the vibrational changes among components in the film caused by functional groups. As depicted in Figure 1. FTIR analysis revealed distinct structural differences between the control film (CS) and PL-incorporated composite films. These spectral shifts and additional bands are consistent with those reported in the literature for chitosan and ε-polylysine conjugates, confirming chemical interactions such as hydrogen bonding and electrostatic association between PL and CS chains (22). The control showed characteristic peaks at ~1,020 and 1,065 cm^−1^, corresponding to C–O–C and C–OH stretching vibrations of polysaccharides. In contrast, PL-containing films (ε-PL-0.1 and ε-PL-0.15) exhibited additional absorption bands at ~1,530–1,537 cm^−1^ and 3,226–3,250 cm^−1^, attributed to amide II (N–H bending and C–N stretching) and N–H stretching, indicating the successful incorporation of PL and potential hydrogen bonding interactions with the matrix. The increasing intensity of these peaks with higher PL content suggests enhanced molecular integration and intermolecular forces. Similar FTIR spectral changes indicating amide bond formation have been observed when ε-PL was incorporated into starch or gelatin matrices, reflecting molecular interaction between –NH_2_ of PL and other polymer groups (26).

The presence of stable C–H stretching bands at ~2,876–2,880 cm^−1^ across all samples confirms the retention of alkyl chain structures. These results align with previous findings in CS/PL/dextran systems where peak broadening at 1,600–1,700 cm^−1^ was associated with Schiff base linkage and increased hydrogen bonding (27). Overall, PL incorporation modified the molecular configuration of the films, potentially improving their antibacterial activity, thermal stability, and water vapor resistance through enhanced hydrogen bonding and electrostatic interactions.

### 3.3 SEM

**Figure 2:**
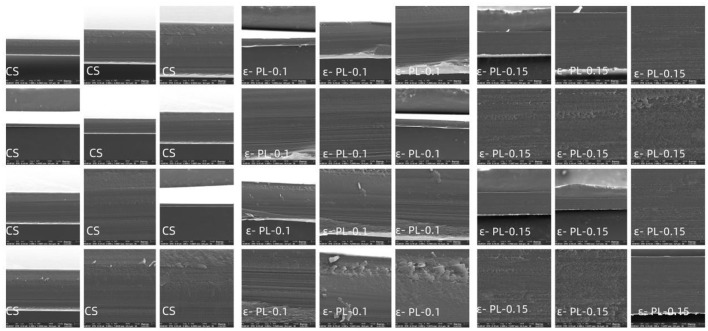
SEM images of different cross-sections of thin films.

Figure 2 shows the cross-sections of each group of films using SEM. The internal microstructure of the films was investigated using scanning electron microscopy to examine the impact of poly-L-lysine (PL) incorporation on the structural organization of the film matrix. The control film (CS) exhibited a rough and stratified cross-sectional profile, with visible layering and irregular voids, indicating a heterogeneous structure with limited internal cohesion. Xu et al. (23) noted that ε-PL acted as a structural crosslinker in CS-based films, enhancing integrity and reducing porosity through electrostatic interactions. In contrast, the cross-section of the PL-0.1 film displayed enhanced structural continuity, with reduced porosity and more compact internal layers. Further incorporation of PL at a concentration of 0.15 led to a markedly dense and homogeneous morphology, with no detectable pores or separations. The improved microstructural integrity can be attributed to strong hydrogen bonding and electrostatic interactions between the cationic PL and polar groups in the polysaccharide matrix, which facilitated closer packing of polymer chains and reduced free volume during film formation.

This morphological evolution from a porous to a dense network aligns with the observed improvements in fundamental physical properties. The denser and more homogeneous microstructure of PL-containing films effectively reduces oxygen diffusion into the tuna surface. By limiting O_2_ availability, the films inhibit oxidative reactions (e.g., myoglobin oxidation and lipid peroxidation) and suppress the growth of aerobic spoilage bacteria, which are key factors driving tuna quality deterioration. The enhanced compactness of the PL-containing films likely contributed to the increased tensile strength and reduced water vapor permeability, as a denser structure restricts water molecule diffusion and improves load-bearing capacity. Gao et al. (28) also confirmed that increased PL concentration contributes to better structural integration, which in turn enhances mechanical and barrier properties.

### 3.4 XRD analysis

**Figure 3:**
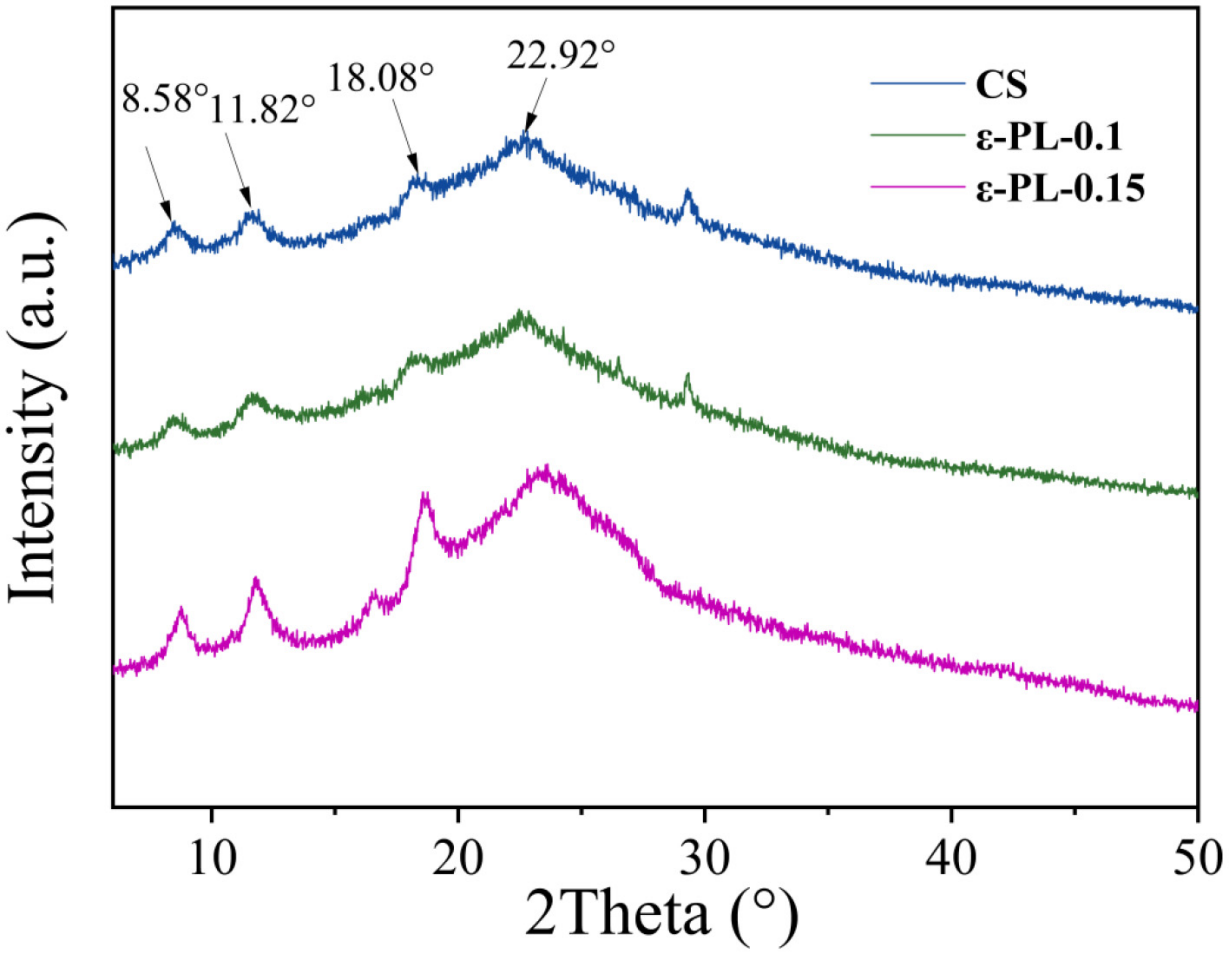
X-ray diffraction patterns of the composite films.

Figure 3 shows the X-ray diffraction patterns of each group of films. The X-ray diffraction patterns of CS and ε-PL composite films show evident changes in crystallinity upon the addition of ε-polylysine. The pure CS film exhibits characteristic diffraction peaks at 2θ ≈ 8.58°, 11.82°, 18.08°, and 22.92°, which are typical of the semi-crystalline nature of chitosan due to its ordered polymer chain packing.

However, with the incorporation of ε-PL (ε-PL-0.1 and ε-PL-0.15), these peaks significantly weaken and broaden, especially at 18.08° and 22.92°, indicating a disruption of the crystalline regions and a transition toward a more amorphous structure. This suggests that strong intermolecular interactions, such as hydrogen bonding or electrostatic forces, between CS and ε-PL interfere with the regular chain alignment.

As ε-PL content increases from 0.1 to 0.15, the diffraction peaks become less defined, further confirming a reduction in crystallinity. The broadening of peaks also reflects enhanced molecular mobility and polymer chain entanglement, which may contribute to better flexibility and structural uniformity. The amorphization facilitated chain entanglement and flexibility, as confirmed by Shih et al. (29), who emphasized the compatibility of PL with hydrophilic polysaccharide matrices.

These changes suggest the successful incorporation of ε-PL into the CS matrix and the formation of a well-integrated composite film. The decrease in crystallinity may also have positive effects on other film properties, including mechanical strength, thermal behavior, and barrier performance. Zhang et al. (30) demonstrated similar crystallinity reduction in CS/PL systems, attributing this to hydrogen bonding and chain disorder. The XRD results demonstrate that ε-PL alters the internal structure of the CS film by reducing crystallinity and promoting the formation of a more amorphous, homogeneous composite network. The reduced crystallinity and more amorphous structure of the composite films are conducive to the migration and release of ε-PL. Unlike the rigid crystalline regions that trap active ingredients, the amorphous matrix provides a more permeable pathway for ε-PL to diffuse to the film-tuna interface, ensuring its effective contact with microorganisms and oxidative substrates, thereby maximizing its antibacterial and antioxidant effects.

### 3.5 Thermal analysis

**Figure 4:**
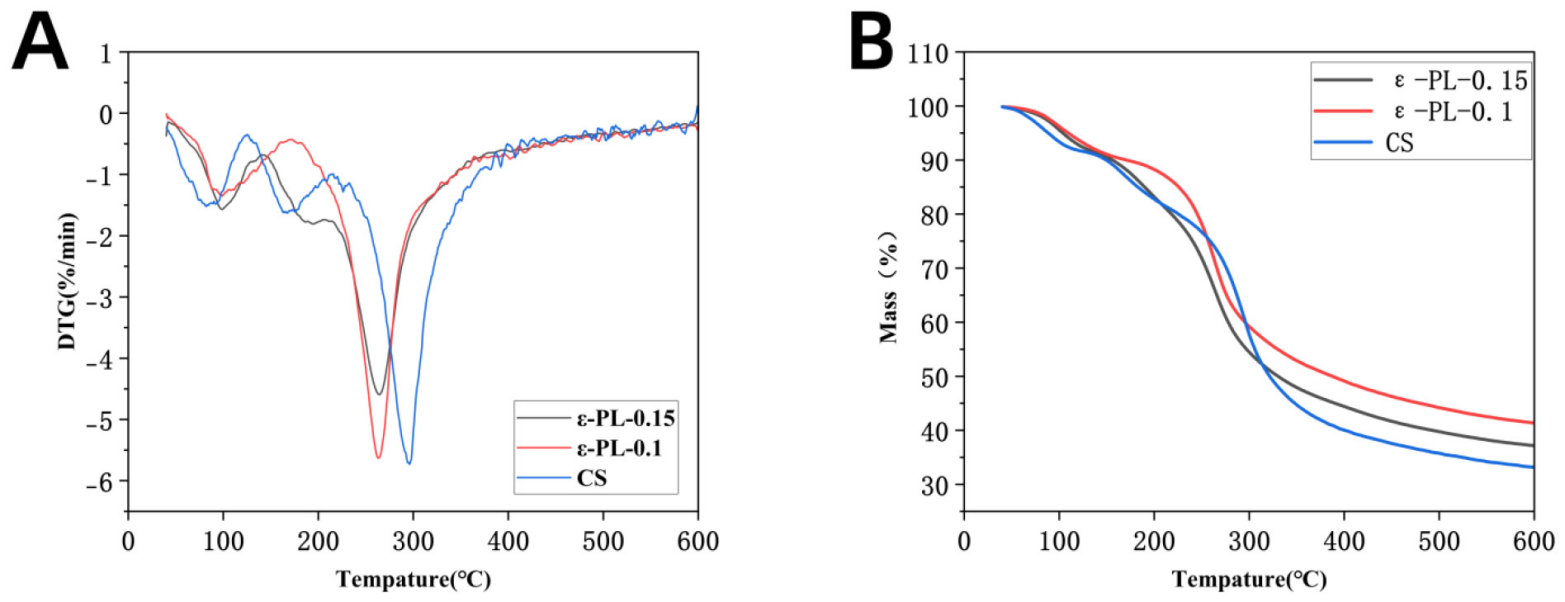
Thermal properties of different films: TG curves **(A)**; DTG curves **(B)**.

TGA is mainly applied to analyze the thermal stability of composite films across a range of temperatures, which plays a key role in ensuring product integrity and preserving important food characteristics, including viscosity and fluidity (31). Figure 4 depicts the thermogravimetric (TG) and derivative thermogravimetric (DTG) curves of each group of thin films. Thermogravimetric analysis demonstrates that polylysine (PL) modification significantly enhances the thermal stability of the films. The CS film exhibits single-stage decomposition beginning at 271.3 °C, with a maximum degradation rate of −5.73%/min at 295.8 °C and a final residue of 33.22% at 600 °C. In comparison, ε-PL-0.15 shows improved thermal resistance despite earlier decomposition onset at 243.3 °C, as evidenced by its reduced maximum degradation rate (−4.59%/min at 264.1 °C) and higher residual mass (37.17% at 600 °C). Chen et al. (32) reported that ε-PL inclusion could introduce additional hydrogen bonding sites, increasing the thermal decomposition temperature of composite fibers.

The DTG curves reveal fundamental differences in degradation mechanisms. While CS displays a single sharp peak at 295.8 °C, ε-PL-0.15 shows two distinct peaks at 194.8 °C and 264.1 °C, indicating the formation of stabilized domains through PL-polymer interactions. The enhanced thermal stability of the composite films ensures structural integrity during food processing (e.g., low-temperature sterilization) and long-term refrigerated storage. By resisting thermal-induced degradation, the film maintains its barrier, controlled-release, and mechanical properties, which are essential for sustained protection of tuna against oxidation and microbial contamination. These include hydrogen bonding between PL amine groups and polymer hydroxyl/acetamido groups, as well as electrostatic interactions. All formulations maintain excellent stability below 100 °C (< 10% weight loss), confirming their processability. The systematic improvement in thermal stability with PL content, measured using NETZSCH TG 209 F1 Libra at 10 °C/min under N_2_ atmosphere, demonstrates PL's effectiveness in creating more thermally resistant film structures while preserving low-temperature performance.

### 3.6 Chilled meat preservation experiment

#### 3.6.1 The change of myoglobin content

**Figure 5:**
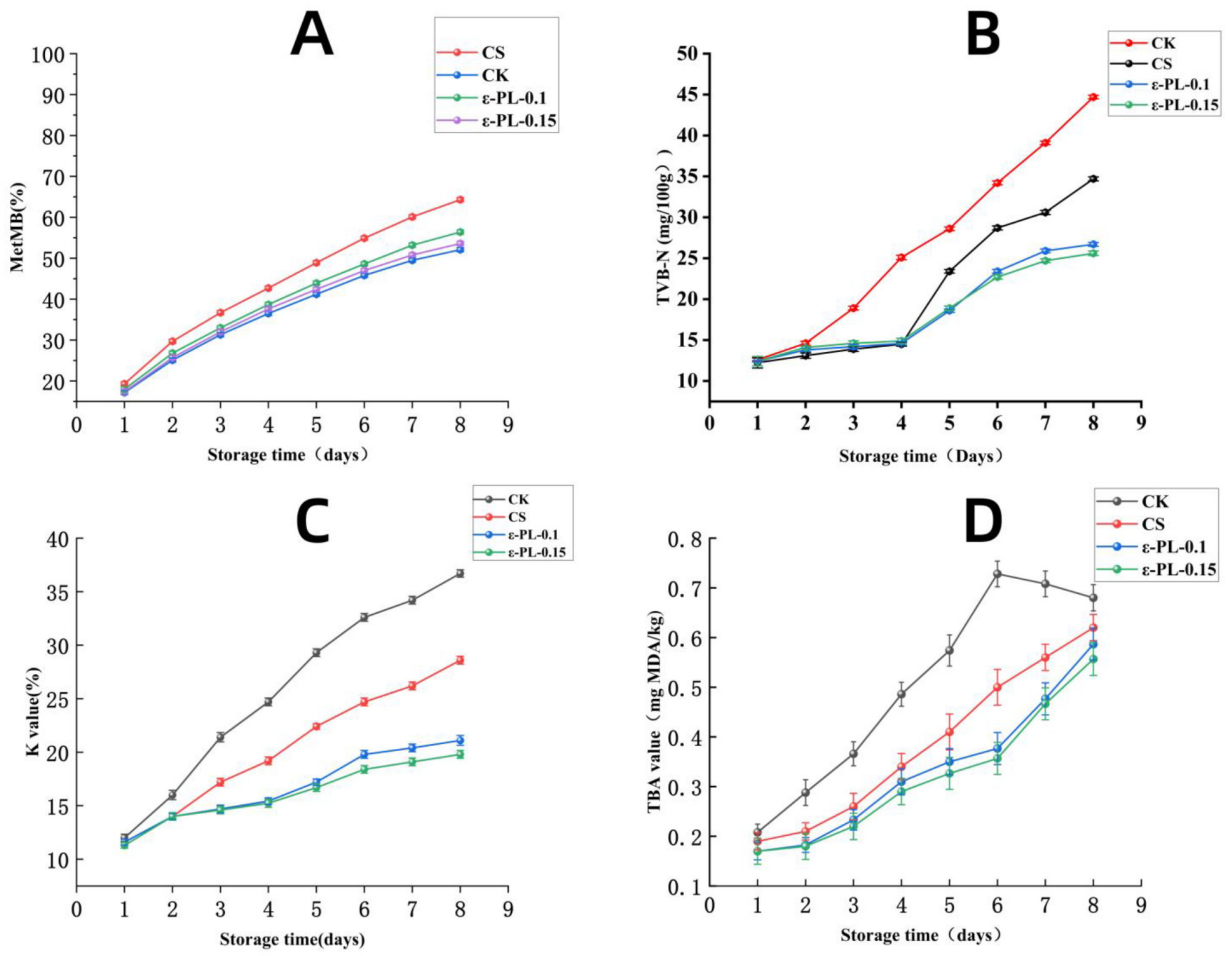
Changes in Metmb **(A)**, TVB-N value **(B)**, *K* value **(C)**, and TBA value **(D)** of samples treated differently during storage at 4 °C.

According to Figure 5A, the extent of myoglobin oxidation is closely related to the loss of freshness and quality in fish meat (3). Previous studies (33–35) have typically established calculation equations based on the absorption spectra of myoglobin in horse hearts (36). This study demonstrates that chitosan–ε-polylysine (ε-PL) composite films effectively preserve the quality of refrigerated yellowfin tuna by simultaneously inhibiting myoglobin oxidation, limiting microbial proliferation, and reducing protein and lipid degradation. Among the tested formulations, the highest ε-PL concentration (ε-PL-0.15) exhibited the most pronounced protective effects, resulting in significantly lower metmyoglobin levels, reduced total volatile basic nitrogen (TVB-N), decreased bacterial counts, and suppressed thiobarbituric acid (TBA) and K values compared to the control. These results indicate that the synergistic action of chitosan and ε-PL not only forms a physical barrier to oxygen and contaminants but also provides antimicrobial and antioxidant activity that stabilizes both surface and internal tissue quality. The concurrent improvement across multiple quality parameters suggests that the composite films mitigate interconnected spoilage pathways, including myoglobin-mediated oxidative reactions and microbial metabolism. This experiment underscores the effectiveness of ε-polylysine in mitigating myoglobin oxidation by scavenging free radicals (37), thereby preserving the fish's freshness (37). Consequently, the films effectively slow the deterioration of color, texture, and flavor, thereby extending the refrigerated shelf life of tuna. Moreover, the use of natural, food-grade biopolymers highlights the practical applicability of this approach as a sustainable alternative to conventional chemical preservatives. Future studies should focus on optimizing the film formulation for industrial-scale application, evaluating its efficacy across different fish species, and assessing performance under variable storage conditions to validate its broader utility in seafood preservation.

#### 3.6.2 TVB-N

The changes in total volatile basic nitrogen (TVB-N) content of tuna under different membrane encapsulation treatments are shown in Figure 5B. TVB-N compounds, including ammonia, trimethylamine (TMA), and dimethylamine (DMA), are produced as a result of microbial activity and the breakdown of proteins and amino acids. These volatile nitrogen compounds are indicative of protein decomposition and microbial growth, both of which are hallmarks of fish spoilage (6). In the current study, the TVB-N values of all four sample groups started at relatively low levels, ranging from 10 to 11 mg/100 g on day 0. However, as the refrigeration period continued, the TVB-N levels in the different treatment groups showed distinct patterns.

The control group exhibited the most significant increase in TVB-N values, with levels rising sharply from 14.6 mg/100 g on day 2 to 44.7 mg/100 g on day 8. This increase is indicative of rapid microbial growth and protein breakdown. The absence of preservatives or antimicrobial agents in this group allowed for the uncontrolled proliferation of spoilage bacteria, leading to the rapid accumulation of volatile nitrogenous compounds. The CS group demonstrated some initial inhibition of TVB-N production, with levels increasing more slowly than the control group. On day 10, the TVB-N value for the CS group was 34.7 mg/100 g, which is still a significant increase but much lower than that observed in the control group. Chitosan has been shown to have some antimicrobial properties. However, the preservation effect of chitosan was not sufficient to completely prevent the rise in TVB-N levels over time.

The ε-polylysine treatment showed the best results in inhibiting TVB-N accumulation. The TVB-N value in the ε-PL-0.1 group was 26.7 mg/100 g by day 8, which is much lower than that in the control and CS groups. The ε-PL-0.15 group exhibited the lowest TVB-N levels. This effectiveness can be attributed to ε-polylysine's strong antimicrobial properties, which inhibit the growth of various spoilage microorganisms, including *B. subtilis, P. fluorescens*, and *Lactobacillus*. By inhibiting these bacteria, ε-polylysine helps to maintain the freshness of the tuna for a longer period.

The composite treatment of ε-polylysine and chitosan (ε-PL and chitosan) exhibited the most remarkable preservation effect. On day 8, the TVB-N value in the composite group was 25.6 mg/100 g. This synergistic effect is likely due to the combination of chitosan's physical barrier properties and ε-polylysine's strong antimicrobial and antioxidant effects. The chitosan membrane physically blocks microbial access to the fish, while epsilon polysaccharide further inhibits microbial growth and oxidation reactions, there is a constitution. A more effective preservation strategy and significantly enhancing the protective performance of the composite coating (38).

#### 3.6.3 *K* value measurement

The trend in the *K* value of yellowfin tuna meat wrapped with different membrane coatings is shown in Figure 5C. The increase in concentration of hypoxanthine riboside (HxR) and hypoxanthine (Hx) reflects the changes in microbial activity and the autolysis stage after fish death (39, 40). Therefore, the *K* value is often used as an indicator of the extent of spoilage in fish and other seafood.

In this study, the *K* values for the different groups increased over time, with the control group showing the largest increase. On day 2, the *K* value for the control group was 16, but by day 8, it had risen to 36.7. The rapid increase in *K* value in the control group suggests that the absence of preservation agents led to accelerated nucleotide breakdown and overall spoilage.

The CS group exhibited a more gradual increase in *K* value, from 16 on day 2 to 27.8 on day 8. This slower increase suggests that chitosan had some preservation effect. However, as with the other parameters, the effect of chitosan alone was not sufficient to fully maintain the freshness of the tuna over the course of the experiment.

The ε-PL-0.1 group showed a smaller increase in *K* value, reaching 24.1 on day 8. Due to its positive charge, polylysine can undergo electrostatic interactions with negatively charged molecules, thereby affecting enzyme activity (41). The ε-PL-0.15 group demonstrated the smallest increase in *K value*, reaching 22.3 on day 8. This result further underscores the superior preservation effect of the composite treatment.

#### 3.6.4 TBA value measurement

Figure 5D illustrates the trend of TBA values for yellowfin tuna meat coated with different film coatings. Lipid oxidation is a major factor contributing to the deterioration of tuna quality during storage, as it leads to the formation of off-flavors, nutrient loss, and the degradation of bioactive compounds (42). In the final stage of automatic oxidation in food oxidation, the main products are TBA reactive substances, which are further oxidized by peroxides to form aldehydes and ketones (43). The thiobarbituric acid (TBA) value, an indicator of malondialdehyde (MDA) content (a secondary product of lipid oxidation), was used to evaluate the antioxidant capacity of different film treatments.

The control group showed a continuous and rapid increase in TBA values throughout the storage period, rising from an initial 0.18 mg/100 g on day 2 to 0.73 mg/100 g on day 8. This significant increase indicates severe lipid oxidation in the untreated tuna, which is consistent with the rapid color degradation observed in Section 3.6.4—both processes are closely associated with oxidative stress during storage. On the 7th day, there was a slight decrease in TBA values in the CK group, which was due to the continued breakdown of MDA as a secondary metabolite of lipid oxidation.

The chitosan (CS) group exhibited a slower rate of TBA value increase compared to the control, reaching 0.61 mg/100 g on day 8. This suggests that chitosan's barrier properties (reducing oxygen penetration) partially inhibit lipid oxidation, as reported in previous studies on seafood preservation (20).

Notably, the ε-polylysine (ε-PL) composite film groups showed superior antioxidant effects. The ε-PL-0.1 group (0.10% ε-PL + 1.5% CS) had a TBA value of 0.58 mg/100 g on day 8, while the ε-PL-0.15 group (0.15% ε-PL + 1.5% CS) exhibited the lowest TBA value (0.55 mg/100 g) among all treatment groups. This concentration-dependent antioxidant effect of ε-PL aligns with its documented activity in inhibiting oxidative reactions in lipid-rich foods (37). The enhanced performance of the ε-PL-0.15 group is attributed to the synergistic effect between chitosan's oxygen barrier and ε-PL's antioxidant activity, which collectively suppresses the progression of lipid oxidation and the formation of oxidative byproducts linked to quality decline.

These results confirm that the CS-ε-PL composite films effectively delay lipid oxidation in refrigerated tuna, with the high-concentration ε-PL formulation (ε-PL-0.15) demonstrating the best protective effect. This is critical for maintaining the sensory and nutritional quality of tuna during extended cold storage.

#### 3.6.5 Microbial count (CFU)

**Figure 6:**
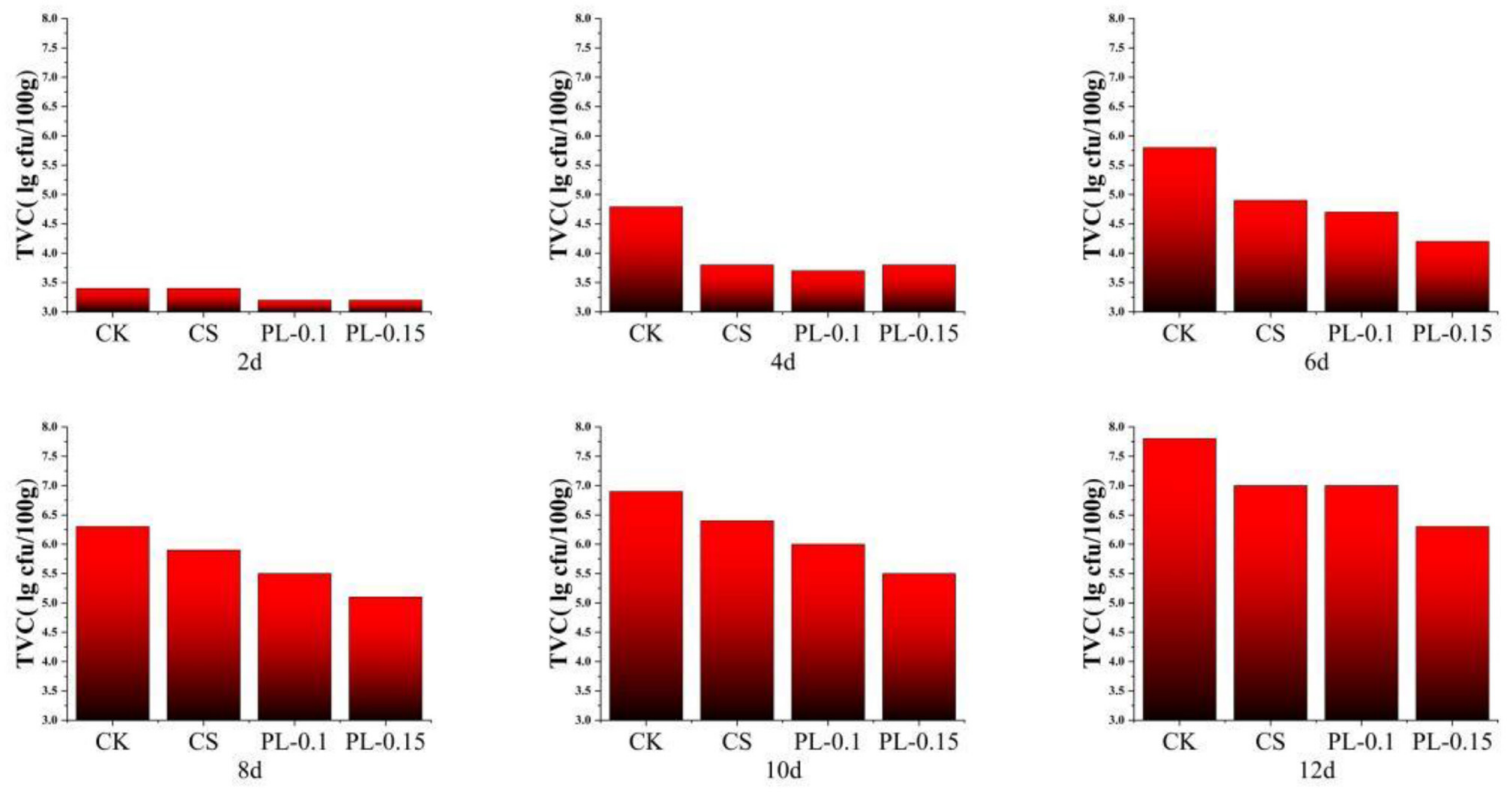
Changes in total bacterial count of samples within 12 days.

The total bacterial count in yellowfin tuna meat wrapped with different films is shown in Figure 6. When the TVC value is below 4 lg (CFU/g), the fish still maintains good edible quality; 5 lg (CFU/g) is its highest acceptable safety standard. When TVC rises to 7 lg (CFU/g), it means that marine fish are no longer suitable for consumption (44). In the current study, the microbial count in the control group increased significantly during storage. By day 12, the microbial count in the control group had reached 7.8 log CFU/g, indicating that microbial growth had advanced to a critical stage. The results from this group underscore the necessity of employing preservation methods to control microbial growth and extend the shelf-life of fish.

In contrast, the chitosan (CS) group exhibited a slower increase in microbial count compared to the control group, reaching 7.0 log CFU/g by day 12. This suggests that chitosan has some inhibitory effects on microbial growth. Despite this, the effect of chitosan alone was not sufficient to prevent microbial growth entirely. The microbial count in the CS group still increased significantly, indicating that while chitosan provides some preservation benefits, its effect is limited, particularly in the absence of other preservatives or longer storage periods.

The groups treated with ε-polylysine (ε-PL), particularly the ε-PL-0.1 and ε-PL-0.15 groups, showed significantly lower microbial counts. By day 12, the microbial count in the ε-PL-0.1 group was 6.8 log CFU/g, while the ε-PL-0.15 group exhibited the lowest microbial count at 6.3 log CFU/g as well. These results suggest that ε-polylysine is highly effective in inhibiting microbial growth. Known for its broad-spectrum antimicrobial activity, ε-polylysine is effective against both Gram-positive and Gram-negative bacteria. The composite treatment group, which combined chitosan and ε-polylysine (ε-PL + CS), exhibited the lowest microbial count among all the treatment groups. On day 12, the microbial count in this composite group was only 6.3 log CFU/g, significantly lower than in any of the other groups. CS, as a cationic polysaccharide, has inherent antibacterial properties (45). Through electrostatic interactions, ε-PL binds to bacterial cell membranes, disrupting their integrity; As a result, the phospholipid bilayer bends and is damaged, causing leakage of cellular contents and ultimately leading to bacterial death (37).

#### 3.6.6 Color measurement (a^*^ value) of tuna

**Figure 7:**
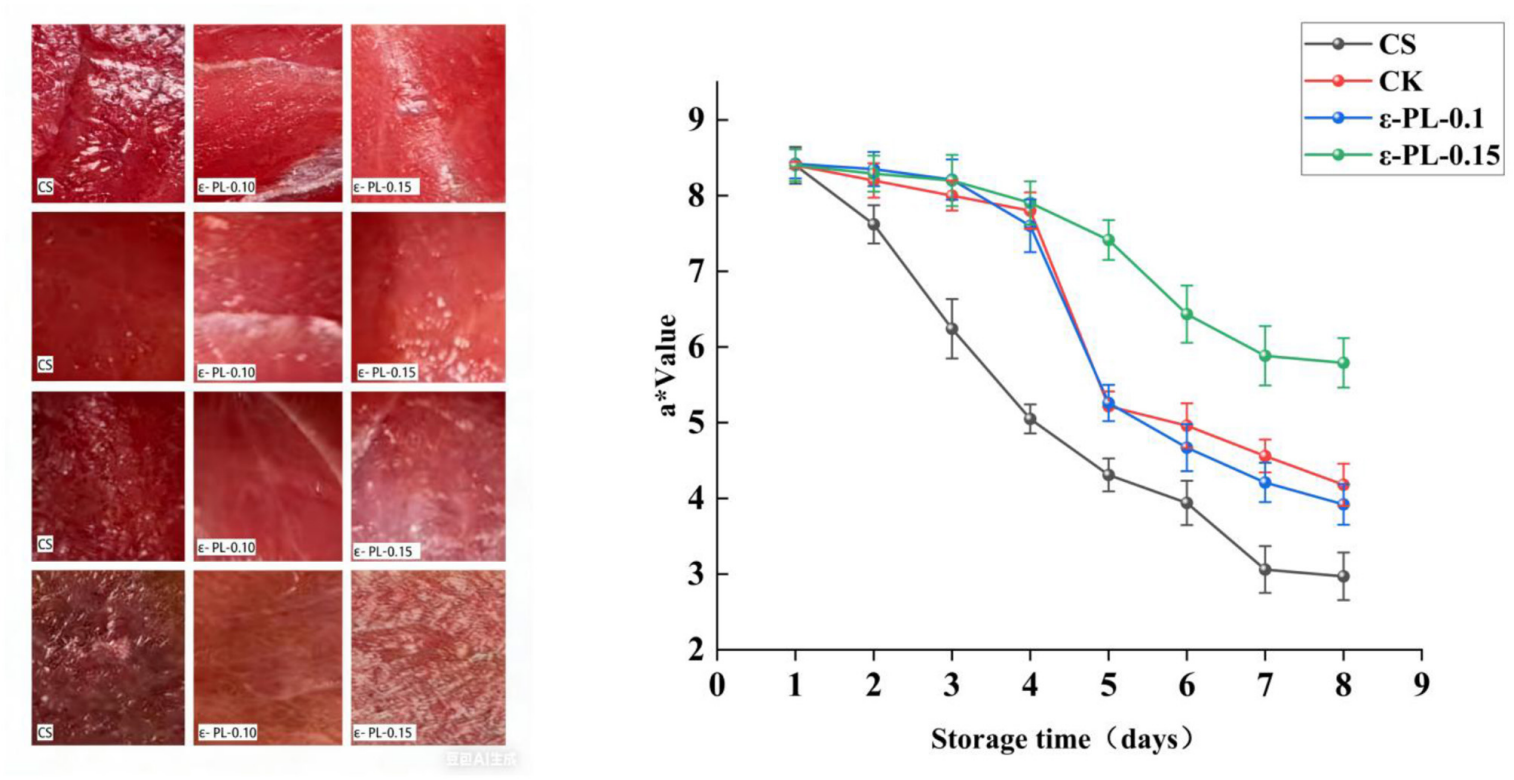
The trend of color difference a value of tuna meat in 1–8 days.

The a^*^ value of yellowfin tuna meat was affected by the different membrane wrapping treatments. Figure 7 shows the changes in the a^*^ value of tuna meat under different membrane wrapping. During storage, the color change of fish meat is influenced by internal and external factors such as pH and temperature, resulting in a decrease in redness and a slight increase in whiteness and yellowness (46). Among the various color parameters, the a^*^ value, which measures the redness of the meat, is commonly employed to assess color preservation during storage. A decrease in the a^*^ value signals a shift from a bright red color, typically associated with freshness, to a more brownish hue, which indicates spoilage and oxidation.

The control group exhibited a rapid decline in a^*^ values, with the initial value of 7.62 on day 2 dropping to 2.97 by day 8. This sharp decrease suggests that the tuna meat in the control group experienced significant oxidation and color degradation, likely due to the breakdown of myoglobin and other heme proteins.

The chitosan (CS) group showed some degree of color preservation, with the a^*^ value decreasing from 8.2 on day 2 to 4.18 on day 8. While the rate of color change was slower compared to the control group, it still indicated that chitosan was not fully effective in preventing oxidation and color degradation.

The ε-polylysine (ε-PL-0.1) group demonstrated a slower decline in a^*^ value compared to the CS group, with the a^*^ value reaching 3.92 on day 8. This indicates that ε-polylysine may possess some ability to delay color change, likely due to its antioxidant properties. ε-Polylysine is known for its ability to scavenge free radicals and inhibit lipid oxidation—two key processes that contribute to the preservation of the red color in fish. The ε-polylysine (ε-PL-0.15) group exhibited the slowest decline in a^*^ value among all treatment groups, with an initial value of 8.29 on day 2 and a value of 5.79 on day 8. The pigments produced by fish after death are generally generated through enzymatic or chemical reactions (47). This result confirms that the composite film with a higher concentration of ε-PL (0.15% ε-PL) is the most effective in preserving the red color of tuna, as it synergistically leverages chitosan's barrier effect and the enhanced antioxidant activity of high-concentration ε-PL to inhibit myoglobin oxidation.

## 4 Conclusion

This study demonstrates that chitosan–ε-polylysine (ε-PL) composite films effectively preserve the quality of refrigerated yellowfin tuna by simultaneously inhibiting myoglobin oxidation, limiting microbial proliferation, and reducing protein and lipid degradation. Among the tested formulations, the highest ε-PL concentration (ε-PL-0.15) exhibited the most pronounced protective effects, resulting in significantly lower metmyoglobin levels, reduced total volatile basic nitrogen (TVB-N), decreased bacterial counts, and suppressed thiobarbituric acid (TBA) and *K* values compared to the control. These results indicate that the synergistic action of chitosan and ε-PL not only forms a physical barrier to oxygen and contaminants but also provides antimicrobial and antioxidant activity that stabilizes both surface and internal tissue quality. The concurrent improvement across multiple quality parameters suggests that the composite films mitigate interconnected spoilage pathways, including myoglobin-mediated oxidative reactions and microbial metabolism. Consequently, the films effectively slow the deterioration of color, texture, and flavor, thereby extending the refrigerated shelf life of tuna. Moreover, the use of natural, food-grade biopolymers highlights the practical applicability of this approach as a sustainable alternative to conventional chemical preservatives. Future studies should focus on optimizing the film formulation for industrial-scale application, evaluating its efficacy across different fish species, and assessing performance under variable storage conditions to validate its broader utility in seafood preservation.

## Data Availability

The datasets presented in this study can be found in online repositories. The names of the repository/repositories and accession number(s) can be found in the article/supplementary material.
